# Role of primary cerebrovascular cilia in the pathogenesis of blood pressure and Alzheimer’s disease manifestations

**DOI:** 10.1002/alz.089289

**Published:** 2025-01-03

**Authors:** Clare Sunderman, Kathleen Forero, Raghad Buqaileh, Wissam Aboualaiwi

**Affiliations:** ^1^ University of Toledo, Toledo, OH USA; ^2^ University of Toledo/College of Pharmacy, Toledo, OH USA

## Abstract

**Background:**

Primary cilia are solitary membrane‐bound organelles emanating from the apical surface of most mammalian cells. They serve as sensory organelles sampling the extracellular environment and reprogramming the transcriptional machinery in response to changes in fluid flow. Ciliopathies, a group of genetic disorders characterized by disrupted cilia structure and/or function, share common phenotypes such as vascular dysfunction and cognitive impairment. Ciliopathies present with a broad range of clinical symptoms, including hypertension (HTN), developmental delays and cognitive and memory deficits, symptoms also observed in Alzheimer’s Disease (AD). This suggests that cilia may house signaling cascades critical for vascular integrity and learning and memory and may also be important contributors to AD and HTN. Therefore, investigation of cilia function in the vascular system may reveal cilia‐modulating mechanisms in neurodevelopmental processes. We propose a bold idea to look at the pathophysiological roles of cerebrovascular ciliary receptors in BP and AD.

**Method:**

Immunofluorescence of human brain endothelial cells (HBECs) were employed to study AChM3R localization and its downstream effectors. Cilia length and function (eNOS activation) studies were carried out to examine if mAChRs activation would enhance cilia structure and function. Vascular‐specific cilia and AChM3R knockout mice were generated to study blood pressure and AD manifestations. Alzheimer’s Disease model mice (3xTg) were compared to AChM3R knockout mice in multiple behavioral studies, Fear Conditioning and Barnes Maze, to look at memory and spacial learning. Control, AChM3R, and 3xTg knockout mouse cerebral arteries were immunostained for various cilia markers.

**Result:**

We discovered that AChM3R, PIP2, and eNOS localize to primary cilia. mAChR‐specific activation increases cilia length in HBECs and cilia formation in cilialess Tg737 cells and was also associated with an increase in eNOS activity. Vascular‐specific AChM3R and Tg737 KO mice developed high BP. Preliminary studies to examine cognitive function indicate altered cognitive function associated with cerebrovascular cilia dysfunction. The two behavioral studies revealed altered memory and fear learning. The 3xTg AD mice cerebral arteries were found to have longer cilia compared to control and AChM3R knockout mice.

**Conclusion:**

Primary cilia play an important sensory role for the regulation of blood pressure and cognitive function.

